# Functional impairment and its associated factors in a working population with bipolar disorder

**DOI:** 10.1192/j.eurpsy.2026.10698

**Published:** 2026-07-31

**Authors:** F. E. Boujmil, G. Bahri, Y. Yaich, G. Amri, Y. Tebourbi, K. Grissa, S. Chemingui, M. Mersni, D. Brahim, M. Bani, R. Ghachem, N. Ladhari

**Affiliations:** 1 Occupational Health department, Charles Nicole Hospital; 2 Psychiatry B department, Razi Hospital, Tunis, Tunisia

## Abstract

**Introduction:**

Bipolar disorder (BD) is commonly associated with a significant functional impairment across multiple domains. Even during euthymic phases, many individuals with BD exhibit deficits in cognitive, social and occupational functioning. These impairments are often under-diagnosed in clinical practice despite their strong association with relapse risk and long-term disability.

**Objectives:**

To assess the overall functional impairment among working patients with BD using a validated questionnaire and to identify its main associated risk factors.

**Methods:**

This cross-sectional study was conducted over a one-year period ( 2024–2025) among consenting patients with BD referred for fitness-for-work evaluation at Charles Nicolle Hospital in Tunisia. Socio-demographic, clinical and occupational data were collected via a self-administered questionnaire. Functional impairment was assessed using the Functioning Assessment Short Test (FAST), a 24-item clinician-rated scale covering six domains: autonomy, occupational functioning, cognitive functioning, financial management, interpersonal relationships, and leisure activities. Each item is scored on a four-point Likert scale, with a total score >11 indicating global functional impairment.

**Results:**

A total of 106 patients were enrolled. The mean age was 43.8 ± 10.3 years with a gender ratio of 0.53 (F/H). Fifty six percent (n=59) were married and had at least one child. The majority had an average socio-economic status (72.6%). Among the patients, 41.5% (n=44) were active smokers and 12.3% (n=13) misused alcohol. The most common occupational categories were nurses (18.9%) and laborers (14.2%). The average length of service was 17.41 ± 8.7 years. Amid the patients, 33.9% (n=36) had atypical working hours. A history of long-term sick leave for psychiatric reasons was present in 88.7% of cases (n=94). More than half of the patients had type 2 BD (64.2%, n = 68) with an average age of onset of 29.7 ± 4.9 years. The mean number of mood episodes was 6.5 ± 3.5. The average length of hospitalization was 6.4 ± 14 days. The majority of patients (n=93, 86.9%) were receiving anticonvulsants and atypical antipsychotics. Good treatment compliance was observed in 73.6% of cases. The mean FAST score was 21,98 ± 8,20. More than threequarters of the patients (89,6%) had a global functional impairment. The most affected domains were occupational functioning (64,2%) and interpersonal relationships (63,2%). Univariate analysis has revealed statistically significant associations between higher FAST scores with gender (p = 0,02) and benzodiazepines use (p = 0,012).

**Conclusions:**

These findings highlight the need for routine functional assessments and considered reducing benzodiazepine use and promoting targeted psychosocial interventions may help improve occupational functioning.

**Disclosure of Interest:**

None Declared

